# Bach1 Inhibition Suppresses Osteoclastogenesis via Reduction of the Signaling via Reactive Oxygen Species by Reinforced Antioxidation

**DOI:** 10.3389/fcell.2020.00740

**Published:** 2020-08-04

**Authors:** Satoshi Wada, Hiroyuki Kanzaki, Yuta Katsumata, Yuuki Yamaguchi, Tsuyoshi Narimiya, Otis C. Attucks, Yoshiki Nakamura, Hiroshi Tomonari

**Affiliations:** ^1^Department of Orthodontics, School of Dental Medicine, Tsurumi University, Yokohama, Japan; ^2^vTv Therapeutics LLC, High Point, NC, United States

**Keywords:** Bach1, osteoclastogenesis, ROS, antioxidant enzymes, Nrf2

## Abstract

Bone destructive diseases such as periodontitis are common worldwide and are caused by excessive osteoclast formation and activation. Receptor activator of nuclear factor-κB ligand (RANKL) is essential factor for osteoclastogenesis. This triggers reactive oxygen species (ROS), which has a key role in intracellular signaling as well exerting cytotoxicity. Cells have protective mechanisms against ROS, such as nuclear factor E2-related factor 2 (Nrf2), which controls the expression of many antioxidant enzyme genes. Conversely, BTB and CNC homology 1 (Bach1), a competitor for Nrf2, transcriptionally represses the expression of anti-oxidant enzymes. Previously, we demonstrated that RANKL induces Bach1 nuclear import and attenuates the expression of Nrf2-mediated antioxidant enzymes, thereby augmenting intracellular ROS signaling and osteoclastogenesis. However, it remains unknown if Bach1 inhibitors attenuate osteoclastogenesis. In this study, we hypothesized that Bach1 inhibition would exert an anti-osteoclastogenic effects via diminishing of intracellular ROS signaling through augmented antioxidation. We used RAW 264.7 cells as osteoclast progenitor cells. Using flow cytometry, we found that Bach1 inhibitors attenuated RANKL-mediated ROS generation, which resulted in the inhibition of osteoclastogenesis. Local injection of a Bach1 inhibitor into the calvaria of male BALB/c mice blocked bone destruction induced by lipopolysaccharide. In conclusion, we demonstrate that Bach1 inhibitor attenuates RANKL-mediated osteoclastogenesis and bone destruction in mice by inducing the expression of Nrf2-regulated antioxidant enzymes that consequently decrease intracellular ROS levels. Bach1 inhibitors have potential in inhibiting bone destructive diseases such as periodontitis, rheumatoid arthritis and osteoporosis.

## Introduction

Osteoclasts are multi-nucleated cells derived from macrophages/monocytes and play a central role in bone resorption (Harada and Rodan, 2003). In bone destructive diseases such as periodontitis, rheumatoid arthritis and osteoporosis, dysregulation of osteoclasts cause bone destruction (Taubman et al., 2005). Several pathways are known to regulate osteoclast differentiation, with macrophage colony-stimulating factor (M-CSF) and Receptor activator of nuclear factor-κB ligand (RANKL) particularly critical for osteoclastogenesis (Takayanagi, 2007). Reactive oxygen species (ROS) is an important signal for osteoclastogenesis to induce the change of the concentration of intracellular calcium ion (Bax et al., 1992). Nuclear factor E2-related factor 2 (Nrf2) is a transcriptional factor which binds to antioxidant response elements (ARE) and controls gene expression of many antioxidant enzymes against oxidative stress (Favreau and Pickett, 1991; Wild et al., 1998; Alam et al., 1999; Thimmulappa et al., 2002). Previously, we reported that overexpression of Nrf2 inhibited osteoclastogenesis, while knockdown of Nrf2 induced osteoclastogenesis (Kanzaki et al., 2013). Therefore, we consider Nrf2 a key regulatory molecule of osteoclastogenesis.

We previously demonstrated that RANKL induces nuclear import of BTB and CNC homology 1 (Bach1) and nuclear export of Nrf2 (Kanzaki et al., 2017). We additionally found that Bach1 is exported from the nucleus to the cytoplasm by 5-aminolevulinic acid (ALA) and sodium ferrous citrate (SFC) treatment, which is followed by an increase in nuclear Nrf2, and inhibition of osteoclastogenesis (Kanzaki et al., 2017). Bach1 is a transcriptional repressor that binds to AREs and suppresses Nrf2 activity (Dhakshinamoorthy et al., 2005). It has been also reported that gold nanoparticles and sodium arsenite elicit Bach1 nuclear export and Nrf2 nuclear import, and increase expression of anti-oxidative enzymes such as NQO1 and HMOX1 (Liu et al., 2013; Lai et al., 2015). While Bach1 nuclear import is considered important for osteoclastogenesis (Kanzaki et al., 2017), it remains unknown whether Bach1 inhibition, which accelerates Bach1 nuclear export, exerts an anti-osteoclastogenic effect.

In this study, we hypothesized that Bach1 inhibition would suppress osteoclastogenesis and bone destruction via attenuation of intracellular ROS signaling through antioxidant mechanisms. To test this hypothesis, we used Bach1 inhibitors for *in vitro* and *in vivo* experiments.

## Materials and Methods

### Chemicals

**FIGURE 1:**
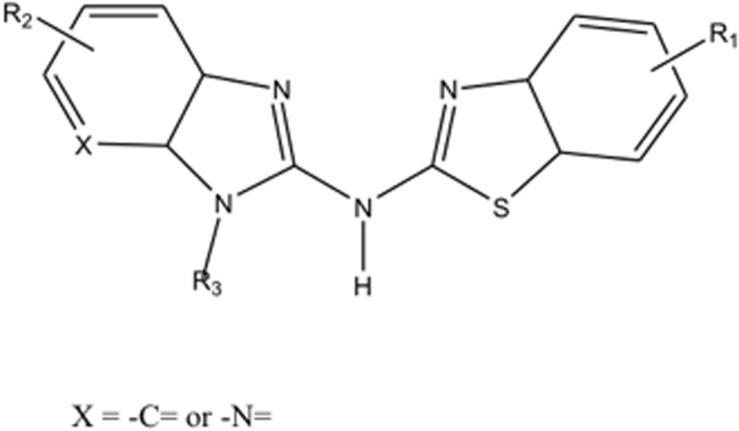
The core structure of compounds HPP-A, HPP-B, HPP-C, and HPP-E.

Recombinant RANKL was purchased from Wako Pure Chemical (Osaka, Japan). Compounds High Point Pharmaceuticals (HPP)-A, HPP-B, HPP-C, and HPP-E are provided by vTv Therapeutics LLC, and each compound is subject to H^1^ NMR analysis for structural and purity (>95%) verification. Compounds HPP-A, HPP-B, HPP-C, and HPP-E each have the same core structure but different sidechain structures (R1 to R3) shown Figure 1 and may be prepared according to the general procedures provided in PCT International Publication Number WO 2011/103018. HPPs are able to modulate the transcriptional repression of Bach1 of ARE genes (Attucks et al., 2014). Potential binding of HPPs to Bach1 were also confirmed. Furthermore, these HPPs are non-electrophilic molecule which have shown not to conjugate with glutathione.

### Cells

Mouse monocyte cell line, RAW264.7, was obtained from the Riken Bioresource Center (Tsukuba, Japan).

### Cell Culture

RAW264.7 cells were cultured in α-modified Eagle’s medium (Wako Pure Chemical) that contained 10% fetal bovine serum (FBS; Atlas Biologicals, Fort Collins, CO, United States), with antibiotics (100 U/mL of penicillin and 100 μg/mL of streptomycin). All cells were cultured at 37°C in a 5% CO_2_ incubator.

### Cell Viability Assay

Cytotoxicity of Bach1 inhibitors was examined using the AlamarBlue^TM^ Cell Viability Reagent (Thermo Fisher Scientific, San Jose, CA, United States). In brief, RAW264.7 cells were seeded on 24-well plates and cultured with various concentrations of Bach1 inhibitors for 1 day. AlamarBlue^TM^ Cell Viability Reagent was added to culture media and fluorescence intensity (excitation: 530 nm, emission: 590 nm) measured using the Synergy HTX Multi-Mode plate Reader (BioTek Japan, Tokyo, Japan) after 1 h.

### Osteoclastogenesis Assay

Cells plated in 96-well plates (RAW264.7 cells: 10^3^ cells/well) in triplicate were stimulated by recombinant RANKL (100 ng/ml) with or without Bach1 inhibitors. Cells were stained for tartrate-resistant acid phosphatase (TRAP) using an acid phosphatase kit (Sigma-Aldrich, St. Louis, MO, United States) after 4 days of culture. Dark red multinucleated cells (≥3 nuclei) were counted as TRAP-positive multinucleated cells.

### Real-Time RT-PCR Analysis

RNA was extracted from RAW264.7 cells using the GenElute mammalian total RNA Miniprep kit (Sigma-Aldrich, St. Louis, MO, United States) with on-column genomic DNA digestion in accordance with the manufacturer’s instructions. For gene expression analysis of antioxidant enzymes, RNA was extracted at 1 day after Bach1 inhibitors treatment, and the gene expressions of *Hmox1* and *Nqo1* was analyzed because these anti-oxidant enzymes were reported to relate osteoclastogenesis (Zwerina et al., 2005; Sakamoto et al., 2012; Hyeon et al., 2013). For gene expression analysis of osteoclast markers, RNA was extracted at 4 days after RANKL stimulation, and the gene expressions of *Atp6v0d2*, *Cathepsin K*, *Matrix metalloproteinase 9*, *Trap*, *Dcstamp*, and *Oscar* were analyzed. RNA (500 ng) was reverse transcribed with iScript cDNA-Supermix (Bio-Rad Laboratories, Hercules, CA, United States).

Real-time RT-PCR was performed with SsoFast EvaGreen-Supermix (Bio-Rad Laboratories). PCR primers used in the experiments were from PrimerBank and are previously described (Kanzaki et al., 2013). Fold changes of gene of interest were calculated by using the Δ-Δ Ct method with *Ribosomal protein S18* as reference gene. Data shown are representative of three independent experiments performed in triplicate.

### Resorption Assay

RAW264.7 cells were plated on synthesized calcium phosphate substrate (bone resorption assay plate; PG Research, Tokyo, Japan) and stimulated with RANKL in the presence or absence of Bach1 inhibitors. After 7 days of cultivation, cells were removed with bleach, and the calcium phosphate substrate was washed with distilled water and then dried. Photographs were taken, and the average of the resorbed area per field was calculated from 12 images of each sample, using the software ImageJ (National Institutes of Health, Bethesda, MD, United States).

### Preparation of Nuclear Protein Lysate

Nuclear protein lysate was prepared from RAW 264.7 cells using the LysoPure^TM^ Nuclear and Cytoplasmic Extractor Kit (Wako, Osaka, Japan) according to the manufacturer’s instructions. Nuclear protein samples were extracted after 6 h of HPP-E treatment. Briefly, cultured cells were washed with PBS and treated with cell lysis buffer. After centrifugation, the nuclear pellet was washed twice and lysed with nuclear lysis reagent. The supernatant was used as the nuclear protein extract after centrifugation. The protein concentrations of the nuclear lysates were measured with the Quick Start^TM^ protein assay kit (Bio-Rad), and concentrations adjusted to be similar.

### Western Blot Analysis

Prepared protein lysates were then electrophoresed on TGX Precast gels (Bio-Rad Laboratories), and proteins were transferred to PVDF membranes using a Trans-Blot^®^ Turbo^TM^ (Bio-Rad Laboratories). After washing, membranes were treated by PDVF Blocking Reagent^®^ (Toyobo Co., Ltd., Osaka, Japan), then incubated with a primary antibody diluted in Can Get Signal^®^ Solution-1 (Toyobo Co., Ltd.). After thorough washing with PBS containing 0.5% of Tween-20 (PBS-T), membranes were incubated with horse-radish peroxidase-conjugated secondary antibody in Can Get Signal Solution-2 (Toyobo Co., Ltd.), and washed with PBS-T. Chemiluminescence was produced using Luminata Forte (EMD Millipore Corporation, Billerica, MA, United States) and detected with LumiCube (Liponics, Tokyo, Japan). Primary antibodies used were anti-Nrf2 (1/1000 dilution; Santa Cruz Biotechnology Inc., Santa Cruz, CA, United States), and anti-histone H3 (1/4000; Cell Signaling Technology Japan, Tokyo, Japan).

### Intracellular ROS Detection

Cells were pretreated with or without HPP-E for 1 h, stimulated with soluble RANKL for 6 h, washed with PBS, and collected. Cells were then stained with fluorescent superoxide probe (BES-So-AM; Wako Pure Chemical) on ice. After washing, intracellular ROS was detected using a flow cytometer (AccuriC6; BD Biosciences, San Jose, CA, United States). The viable monocyte/macrophage fraction was gated on a forward scatter/side scatter plot, and intracellular ROS levels were monitored in the FL-1 channel. Data shown are representative of three independent experiments performed in triplicate.

### *In vivo* Bone Destruction Model

All animals were treated ethically, and animal experiments were performed in compliance with the Regulations for Animal Experiments and Related Activities at Tsurumi University. The calvarial bone destruction mouse model, induced by repeated LPS injections, has been described previously (Kanzaki et al., 2014). Twenty 7-week-old male BALB/c mice (Clea Japan, Tokyo, Japan) were used. Mice were randomly assigned to four groups (*n* = 5 each): a Dimethyl sulfoxide (DMSO)-injected group (control group; 10 μl PBS + 2 μl DMSO), a HPP-E-injected group (HPP-E group; 10 μl PBS + 2 μl of HPP-E 2 μM), an LPS-induced bone resorption group (LPS group; 10 μl of 1 μg/μl LPS + 2 μl DMSO), and an LPS-induced bone resorption and HPP-E-injected group (LPS + HPP-E group; 10 μl of 1 μg/μl LPS + 2 μl of HPP-E 2 μM). To reduce the number of experimental animals, we did not set the group of no-injection or PBS-injection because of the previous experiments revealed no difference of these groups to DMSO-injected group (data not shown).

Under anesthesia, injections were performed with a 30-gauge needle at a point on the midline of the skull located between the eyes on days 1, 3, 5, 7, and 9. On day 11, mice were sacrificed by cervical dislocation and cranial tissue samples were fixed overnight with 4% paraformaldehyde in PBS.

### Micro-Computed Tomography Analysis for Bone Destruction

Samples of cranial tissue were scanned with an X-ray micro-computed tomography (μCT) system (inspeXio SMX-225CT; Shimadzu Corp., Kyoto, Japan). After reconstitution, the DICOM files were rendered into three-dimensional images using Pluto software (Kanzaki et al., 2014). Percentage of resorbed area, calculated from the ratio of the number of pixels in the resorbed area in the cranial bone to the number of pixels in the total analyzed image of the cranial bone, was calculated with ImageJ software. The region of interest was set between the fronto-parietal (coronal) suture and parieto-occipital (lambdoidal) suture.

### Statistical Analysis

All data are presented as the mean ± standard deviation from three independent experiments. ANOVA and Tukey’s HSD test were used for evaluating the statistical significance (SPSS^®^ 11.0J; IBM, Chicago, IL, United States). *P* < 0.05 was considered to be statistically significant.

## Results

### Assessment of Bach1 Inhibitor Cytotoxicity

**FIGURE 2:**
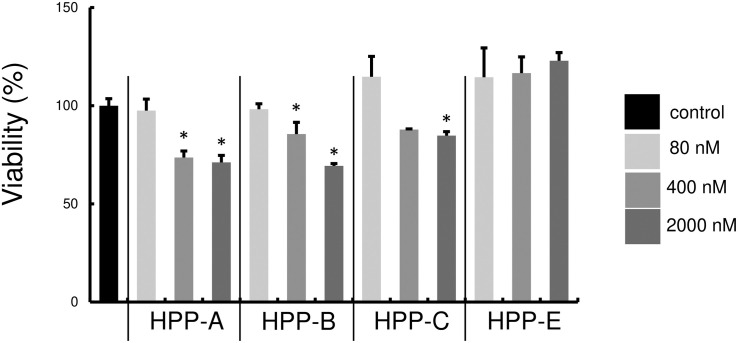
Cytotoxicity of Bach1 inhibitors against RAW 264.7 cells. Cytotoxicity at various concentrations of HPPs was assessed using AlamarBlue reagent. Percentage of control is shown. The data shown are representative of three independent experiments performed in triplicate. ^∗^*P* < 0.05 vs. control.

First, we examined whether Bach1 inhibitors, HPP-A, HPP-B, HPP-C, and HPP-E, exhibit cytotoxicity against RAW264.7 cells. There was no cell cytotoxicity at 80 nM, but the highest tested concentration of 2000 nM exhibited cytotoxicity compared with the control (Figure 2). Therefore, we used 80 and 400 nM of HPPs for subsequent experiments due to no cytotoxicity of all HPPs at 80 nM, and relatively low level of cytotoxicity at 400 nM.

### Bach1 Inhibitors Suppress RANKL-Mediated Osteoclastogenesis

**FIGURE 3:**
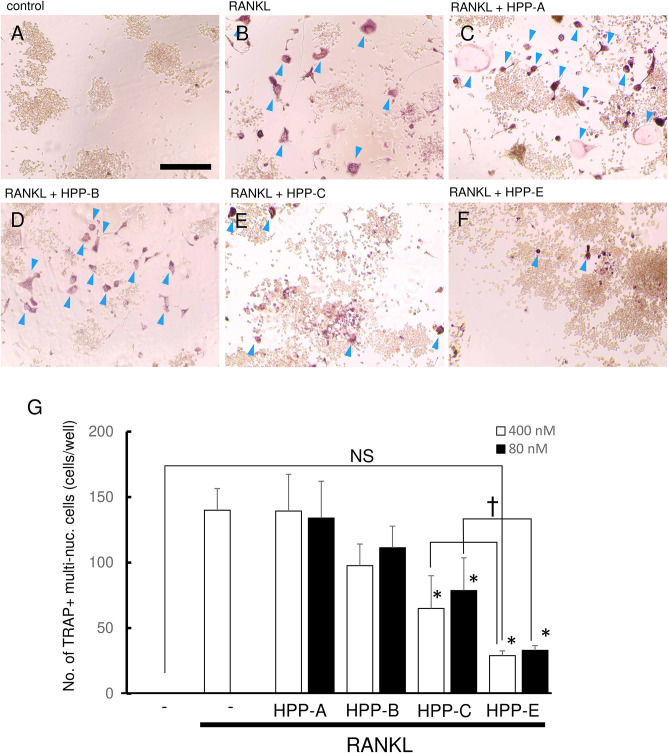
Bach1 inhibitors attenuate RANKL-mediated osteoclastogenesis. Representative photographs of TRAP staining are shown of randomly selected field: **(A)** control, **(B)** RANKL (100 ng/ml), **(C)** RANKL (100 ng/ml) + HPP-A (400 nM), **(D)** RANKL (100 ng/ml) + HPP-B (400 nM), **(E)** RANKL (100 ng/ml) + HPP-C (400 nM), and **(F)** RANKL (100 ng/ml) + HPP-E (400 nM). Arrowhead indicates TRAP-positive multinucleated cells. Scale bars; 100 μm. **(G)** Mean number of TRAP-positive multi-nucleated cells differentiated from RAW 264.7 cells at 400 nM (open bar) and 80 nM (close bar). The data shown are representative of three independent experiments performed in triplicate. ^∗^*P* < 0.05 vs. RANKL alone. ^†^*P* < 0.05 between the groups. NS: no significant difference compared with the control.

We next examined whether Bach1 inhibitors exhibited an inhibitory effect on RANKL-dependent osteoclastogenesis. RANKL (100 ng/ml) stimulation of RAW264.7 cells gave a high number of TRAP-positive multinucleated cells, compared with the control (Figures 3A,B). Even at 400 nM, HPP-A and HPP-B had no effect on the number of TRAP-positive multinucleated cells (Figures 3C,D), but they decreased following treatment with HPP-C, and HPP-E (Figures 3E,F). The size and the number of nuclear in the multi-nucleated cells seemed to be reduced by the treatment of HPPs. The comparison at lower concentration (80 nM) clearly demonstrated the stronger inhibition of osteoclastogenesis of HPP-E as compared to HPP-C. Among them, HPP-E resulted in a decrease compared to controls (Figure 3G). These results indicated that HPP-E strongly inhibits osteoclast differentiation in RAW264.7 cells *in vitro*.

### Bach1 Inhibitors Suppress Osteoclast Function

**FIGURE 4:**
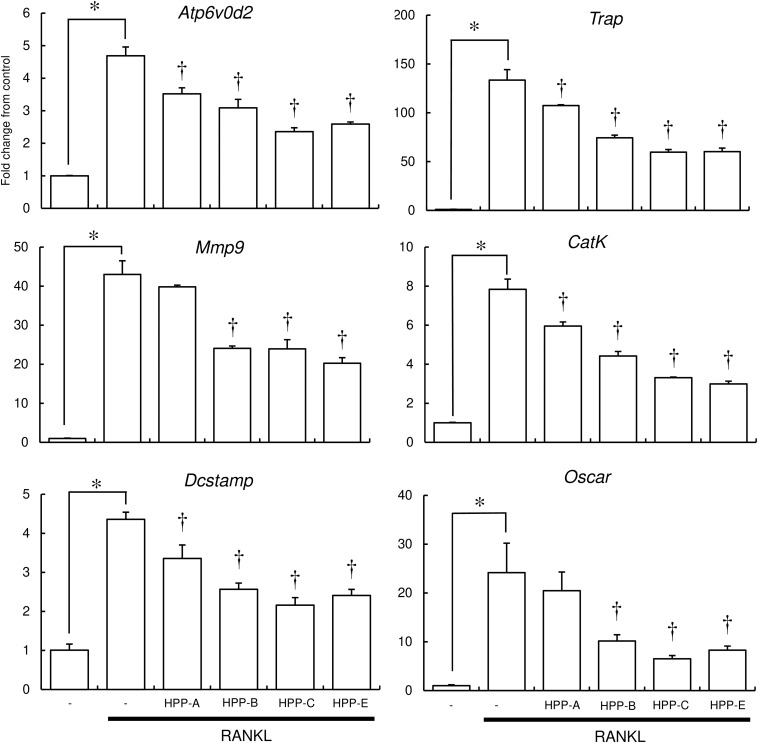
Real-time PCR analysis of osteoclast marker gene expression. The expression of osteoclast marker genes (*Atp6v0d2, Trap, Mmp9, Cathepsin K, Dcstamp, and Oscar*) in RANKL-stimulated RAW 264.7 cells were examined by real-time PCR. Gene expression was calibrated using the *Rps18* house-keeping gene, and values indicate the fold-change compared with control. Data shown are representative of three independent experiments performed in triplicate. ^∗^*P* < 0.05 vs. control. ^†^*P* < 0.05 vs. RANKL alone.

To further examine the inhibitory effect of Bach1 inhibitors on osteoclastogenesis, the expression of several osteoclast differentiation marker genes in RAW264.7 cells was examined using real-time RT-PCR. RANKL induced the expression of osteoclast marker genes, which, in turn, was suppressed by HPP-B, HPP-C, and HPP-E (Figure 4). HPP-A showed weak suppression of these marker genes, with no significant differences in *Atp6v0d2*, *Mmp9*, and *Oscar*.

**FIGURE 5:**
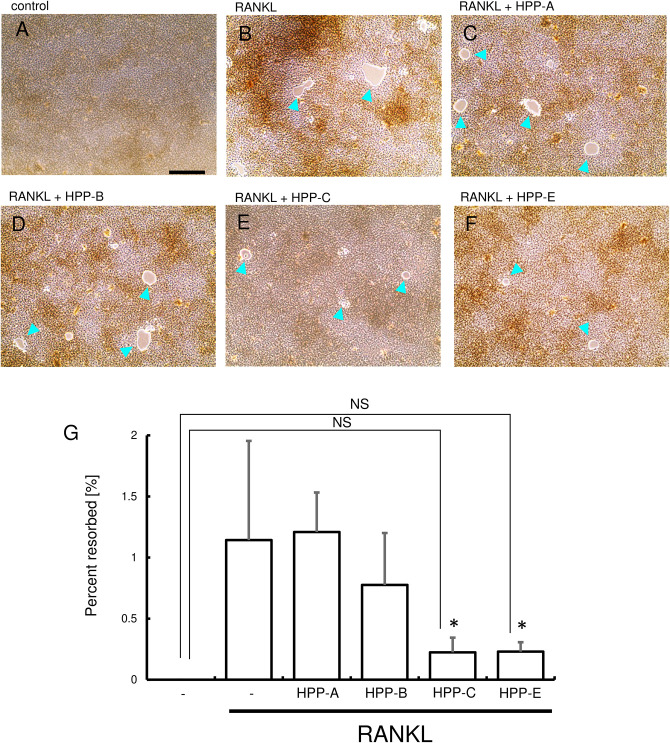
Resorption assay. RAW 264.7 cells were treated with Bach1 inhibitors following stimulation with RANKL (100 ng/mL). Representative photographs are shown of randomly selected fields: **(A)** control, **(B)** RANKL (100 ng/mL), **(C)** RANKL (100 ng/mL) + HPP-A (400 nM), **(D)** RANKL (100 ng/mL) + HPP-B (400 nM), **(E)** RANKL (100 ng/mL) + HPP-C (400 nM), and **(F)** RANKL (100 ng/mL) + HPP-E (400 nM). Arrowhead indicates resorbed area. Scale bars; 100 μm. **(G)** Percentage resorbed area is shown. The data shown are representative of three independent experiments performed in triplicate. ^∗^*P* < 0.05 vs. RANKL alone. NS: no significant difference against the control.

Next, resorption activity was examined using the bone resorption assay plate (Figure 5). RANKL stimulation of RAW264.7 cells induced numerous resorption areas on the substrate (Figure 5B), and HPP-C and HPP-E reduced the resorption areas (Figures 5E–G). These results suggested that HPP-E inhibited not only osteoclastogenesis, but also osteoclast activity.

### Bach1 Inhibitor Increases Nuclear Nrf2

**FIGURE 6:**
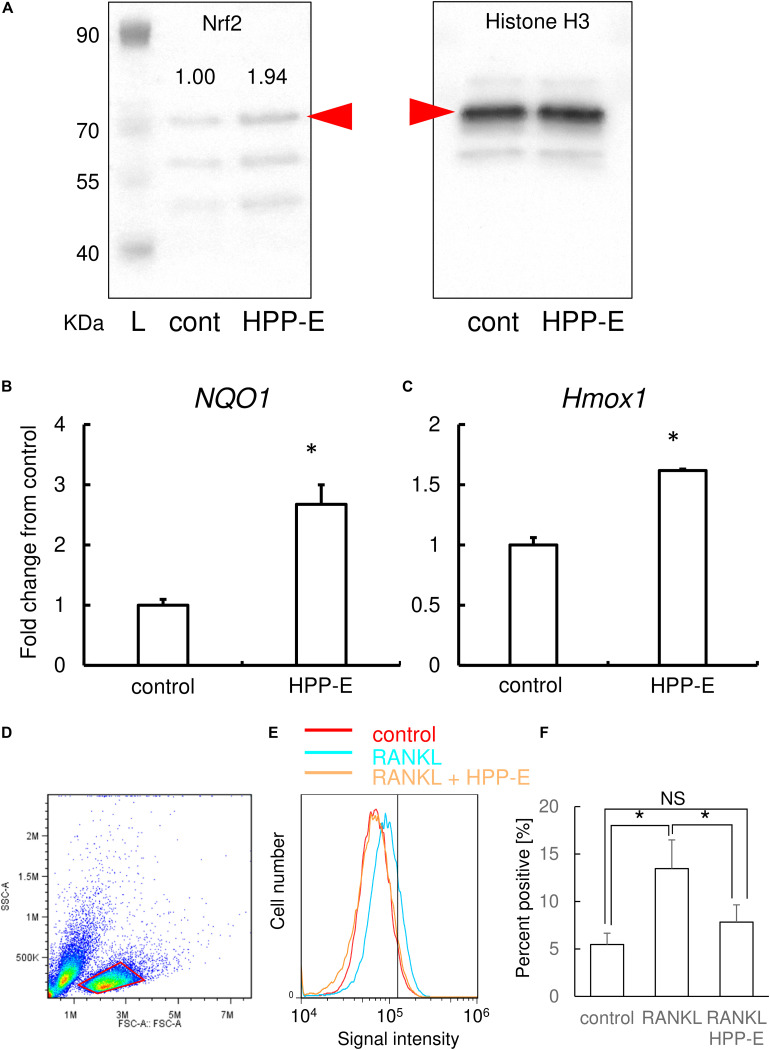
HPP-E induces Nrf2 nuclear translocation, anti-oxidation, and thereby ROS elimination. **(A)** Representative images of western blot analysis of nuclear protein for Nrf2 (the left side panel) and histone H3 (the right side panel) from three independent experiments are shown. Relative band density of Nrf2 normalized by Histone H3 are shown above the each band. Red arrowhead indicates the predicted molecular weight. L: molecular weight markers. Gene expression for *NQO1* **(B)** and *HMOX1* **(C)** are shown. Gene expression was calibrated using the *Rps18* house-keeping gene, and values indicate the fold-change compared with control. The data shown are representative of three independent experiments performed in triplicate. ^∗^*P* < 0.05 vs. control. **(D)** The viable cellular fraction of monocytes/macrophage was gated on a forward scatter/side scatter plot (red area). **(E)** Representative results of intracellular ROS levels in control (red), RANKL-treated (blue), and RANKL- and HPP-E-treated (orange) RAW 264.7 cells from three independent experiments are shown. The vertical line indicates a conventional threshold for ROS-negative and -positive populations. **(F)** Mean percent of ROS-positive RAW 264.7 cells in each groups. ^∗^ *P* < 0.05 versus control.

Based on the results of TRAP staining, real-time RT-PCR, and the bone resorption assay, HPP-E exhibited the most potent inhibition of osteoclastogenesis. Therefore, we examined the molecular mechanism of HPP-E inhibition on osteoclastic signaling. Western blotting using nuclear extracts of RAW264.7 cells clearly demonstrated that treatment of RAW264.7 cells with HPP-E led to nuclear translocation of Nrf2 (Figure 6A). Relative band intensity of Nrf2 calibrated by Histone H3 was almost twice by HPP-E treatment.

### Bach1 Inhibition Augments the Expression of Antioxidant Enzymes

To further examine the effect of HPP-E on the antioxidant response, we examined the expression of antioxidant enzyme genes, such as *Nqo1* and *Hmox1*. The expression of these genes were increased by HPP-E in RAW264.7 cells (Figures 6B,C). This result indicated that HPP-E substantially induces an antioxidant response in RAW264.7 cells.

### Bach1 Inhibition Attenuates RANKL-Mediated Intracellular ROS

Next, we investigated whether HPP-E could interfere with RANKL-triggered intracellular ROS production in RAW264.7 cells. Scatter plot of RAW 264.7 cells gave two populations (Figure 6D). Previously, we compared two population from the point of RANKL-dependent intracellular ROS signaling, and found that the right side population which we set the gate in the present study exhibited responsive ROS signaling, though the left side population exhibited less response (data not shown). Therefore, we set the gate on the right side population in the present study. Stimulation of RAW264.7 cells with RANKL increased intracellular production of superoxide, as detected using BES-So-AM. HPP-E treatment inhibited RANKL-mediated intracellular ROS augmentation (Figures 6E,F), indicating that HPP-E attenuated RANKL signaling by decreasing superoxide production.

### Local Injection of Bach1 Inhibitor Ameliorates RANKL-Dependent Bone Destruction in Mice

**FIGURE 7:**
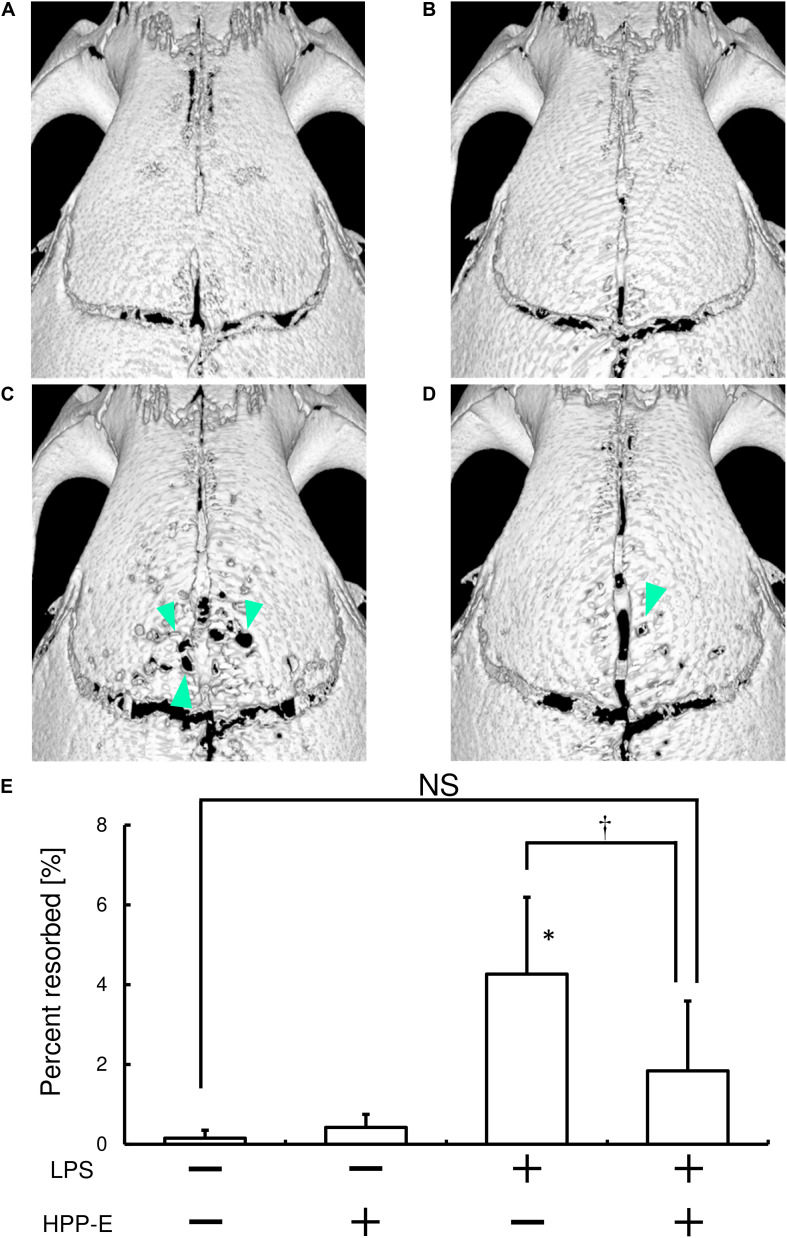
Local HPP-E injection ameliorates LPS-induced bone destruction in mice. Representative μCT images of control **(A)**, HPP-E **(B)**, LPS **(C)**, and LPS+HPP-E treated mice **(D)** are shown. **(E)** Percentage of resorbed area in the cranial bone. NS: no significant difference between the groups. ^∗^*P* < 0.05 vs. control. ^†^*P* < 0.05 between the groups.

Finally, we tested whether local HPP-E injection can ameliorate LPS-mediated bone destruction in mice calvaria. Repeated LPS injection resulted in bone destruction, as compared with the control group (Figures 7A,C). Furthermore, injection of HPP-E had no effect on bone destruction (Figure 7B). μCT imaging of resorbed areas in calvaria clearly demonstrated that the local HPP-E injection ameliorated LPS-mediated bone destruction (Figures 7C,D). We measured resorbed areas and found that HPP-E almost completely inhibited LPS-mediated bone destruction (Figure 7E). These results suggested that HPP-E is a potential inhibitor against bone destruction.

## Discussion

In the present study, we demonstrate for the first time that Bach1 inhibition augmented Nrf2 activation, which resulted in the inhibition of osteoclastogenesis. Bach1 inhibitors increased expression of cytoprotective enzymes, such as HMOX1 and NQO1, and reduced RANKL-mediated ROS generation in RAW 264.7 cells. Furthermore, an *in vivo* mouse bone destruction model demonstrated that a Bach1 inhibitor suppressed LPS-mediated bone destruction. These results indicated that Bach1 inhibitors would be useful for bone destruction diseases like osteoporosis, periodontitis, and rheumatoid arthritis. As to the experimental animal model, we utilized LPS-mediated bone destruction, which induces upregulation of inflammatory cytokine, but also augments RANKL expression (Narimiya et al., 2019). We presumed the animal model in the present study is sufficient to observe the effect of HPP-E on RANKL-mediated osteoclastogenesis *in vivo*.

RANKL-mediated osteoclastogenesis uses ROS for intracellular signaling (Ha et al., 2004), which signifying that the interference against intracellular ROS would be possible anti-osteoclastogenic activity. Indeed, we reported that the activation of anti-oxidation via activation of Nrf2-mediated anti-oxidative enzymes clearly inhibited osteoclastogenesis (Kanzaki et al., 2013, 2014, 2015, 2017; Katsumata et al., 2018; Yamaguchi et al., 2018). Another groups also reported anti-oxidant Quercetin exhibited relevant anti-oxidant properties by high radical scavenging activity, and inhibited osteoclastogenesis (Forte et al., 2017). Taken together, the interference against intracellular ROS can be an anti-osteoclastogenic activity.

Reactive oxygen species causes several diseases by cytotoxic effects, such as peroxidation of lipids and oxidative damage to protein and DNA (Esterbauer et al., 1991; Wells et al., 2009). Cells have several protective mechanisms against this oxidative stress (Kensler et al., 2007). A major one is the transcriptional factor Nrf2, which induces antioxidant enzymes, such as HMOX1, NQO1, and GCS (Favreau and Pickett, 1991; Wild et al., 1998; Alam et al., 1999), making activation of Nrf2 a promising molecular drug target for several diseases (Kwon et al., 2007; Eggler et al., 2008). We previously reported that RANKL induced the nuclear translocation of Bach1 and decreased nuclear Nrf2, attenuated Nrf2-mediated expression of antioxidant enzymes, and augmented osteoclastogenesis (Kanzaki et al., 2017).

Bach1 is a member of the BTB and CNC transcriptional regulator family that binds to ARE sequences as heterodimeric complexes with small Maf proteins and suppresses antioxidant enzyme genes by binding to their ARE sequences (Oyake et al., 1996). Therefore Bach1 and Nrf2 compete with each other to occupy ARE sequences (Suzuki et al., 2003). A previous study demonstrated that the Bach1 inhibitor, HPP-4382, increased Nrf2-imediated antioxidant enzyme genes, such as *HMOX1* and *NQO1* in normal human lung fibroblasts (Attucks et al., 2014). HPPs were able to modulate the transcriptional repression of Bach1 of ARE genes by binding of HPPs to Bach1. We previously also reported that induction of nuclear export of Bach1 by the combination of ALA and SFC increased nuclear transport of Nrf2, which augmented antioxidant enzyme genes. Furthermore, we reported that the induction of nuclear export of Bach1 augmented nuclear translocation of Nrf2 detected by immunofluorescent analysis and western blotting using nuclear protein samples. Bach1 inhibition by ALA/SFC augmented anti-oxidation and inhibited RANKL-mediated osteoclastogenesis from primary peritoneal macrophages. Consistent with these reports, our present study also demonstrated that a Bach1 inhibitor, HPP-E, increased the expression of *HMOX1* and *NQO1* in RAW 264.7 cells. Inflammatory stimulation has been reported to induce Bach1 expression, which promotes attenuation of HMOX1 in leukemic cells (Miyazaki et al., 2010). Furthermore, Bach1-deficient mouse bone marrow-derived macrophages were resistant to RANKL-dependent HMOX1 reduction and impaired osteoclastogenesis (Hama et al., 2012). These findings concur with our observations that Bach1 inhibitors increase the expression of HMOX1 and NQO1 in osteoclast precursors, attenuate RANKL-mediated intracellular ROS signaling, and thereby inhibit osteoclastogenesis. Further confirmatory experiments using primary macrophages are necessary whether Bach1 inhibition by HPPs inhibit osteoclastogenesis.

Various molecules can activate Nrf2 (Jana and Mandlekar, 2009; Nakamura and Miyoshi, 2010; Dao et al., 2015), with dimethyl fumarate (DMF) being one of the most well-known (Talalay et al., 1988; Nelson et al., 1999; Lehmann et al., 2007; Ooi et al., 2011; Yore et al., 2011). Kelch-like ECH-associated protein 1 (Keap1) negatively regulates Nrf2-dependent transcription of cytoprotective enzymes by inhibiting nuclear translocation of Nrf2. DMF protects Nrf2 from ubiquitination and degradation triggered by Keap1 via alkylation of Keap1, which subsequently activates the Nrf2-mediated gene expression. We previously reported that DMF also reduced RANKL-mediated intracellular ROS generation, which resulted in the inhibition of osteoclastogenesis (Yamaguchi et al., 2018). Our present study demonstrated that some HPPs exhibited cytotoxicity except for HPP-E. Anti-osteoclastogenic activity of HPP-A, -B, and -C might partly by this cytotoxicity. HPPs have the same core structure but each HPPs exhibited different property from the point of anti-osteoclastogenic activity and cytotoxicity. We are now investigating how the difference of the side chain structures affect anti-osteoclastogenic activity and cytotoxicity. We previously reported that the cytotoxicity of DMF for RAW 264.7 cells were seen at 100 μM, though HPPs-A, -B, and -C exhibited cytotoxicity even at 400 nM. Further investigations are required to directly compare Bach1 inhibitors and DMF in their effect on osteoclastogenesis.

As to the possible affected stage by HPPs during osteoclastogenesis, we presumed the early stage would be affected due to the reduction of the size and nuclear in the multi-nucleated cells under HPPs treatment, and the reduction of DC-STAMP, which is known as cell-cell fusion molecule during osteoclastogenesis (Kukita et al., 2004), were significantly reduced by HPPs, especially by HPP-E.

**FIGURE 8:**
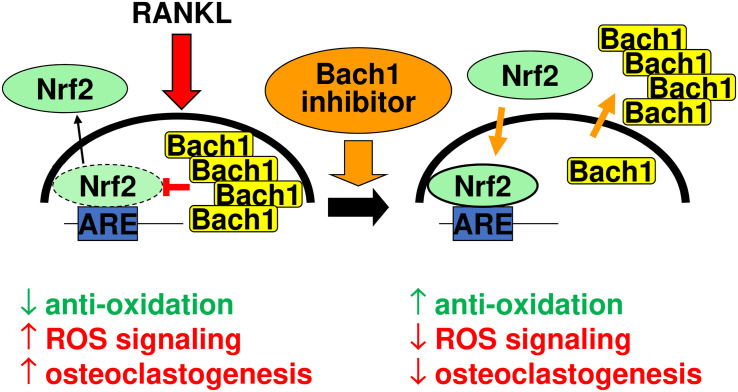
Interpretative competitive regulatory mechanism by Bach1 and Nrf2 during osteoclastogenesis. RANKL stimulation gives rise to induce nuclear translocation of Bach1, which antagonizes to Nrf2 and exclude nuclear Nrf2, promoting osteoclastogenesis by downregulating the anti-oxidation. Bach1 inhibitor promotes nuclear export of Bach1, and induces Nrf2 nuclear accumulation, which augments anti-oxidation that results in the inhibition of osteoclastogenesis.

Figure 8 summarizes our interpretative competitive regulatory mechanism by Bach1 and Nrf2 during osteoclastogenesis. RANKL stimulation gives rise to induce nuclear translocation of Bach1, which antagonizes to Nrf2 and excludes nuclear Nrf2, promoting osteoclastogenesis by downregulating the anti-oxidation. Bach1 inhibitor promotes nuclear export of Bach1, and induces Nrf2 nuclear accumulation, which augments anti-oxidation that results in the inhibition of osteoclastogenesis.

In conclusion, we have demonstrated that Bach1 inhibitor suppressed RANKL-mediated osteoclastogenesis and bone destruction via attenuation of intracellular ROS signaling by augmenting antioxidant enzymes. Therefore, Bach1 inhibitors have potential utility in bone destructive diseases, such as osteoporosis, periodontitis, and rheumatoid arthritis.

## Data Availability Statement

The datasets generated for this study are available on request to the corresponding author.

## Ethics Statement

The animal study was reviewed and approved by the Institutional Animal Care and Use Committee, Tsurumi University (approval number; 28A030).

## Author Contributions

SW and HK wrote the main manuscript text. OA, YN, and HT reviewed and modified the manuscript text. SW, YK, YY, and TN performed the experiments. SW, HK, and OA prepared the figures. All authors reviewed the manuscript.

## Conflict of Interest

OA was employed by company vTv Therapeutics LLC. The remaining authors declare that the research was conducted in the absence of any commercial or financial relationships that could be construed as a potential conflict of interest.
